# Cardiorespiratory Fitness and Physical Activity in Luo, Kamba, and Maasai of Rural Kenya

**DOI:** 10.1002/ajhb.22303

**Published:** 2012-07-27

**Authors:** DL Christensen, D Faurholt-Jepsen, MK Boit, DL Mwaniki, B Kilonzo, I Tetens, FK Kiplamai, SC Cheruiyot, H Friis, K Borch-Johnsen, NJ Wareham, S Brage

**Affiliations:** 1 Department of International Health, University of CopenhagenCopenhagen, Denmark; 2 Steno Diabetes CenterGentofte, Denmark; 3 Department of Human Nutrition, University of CopenhagenFrederiksberg, Denmark; 4 Department of Exercise, Recreation, and Sport Science, Kenyatta UniversityNairobi, Kenya; 5 Center for Public Health Research, KEMRINairobi, Kenya; 6 Division of Nutrition, National Food Institute, Technical University of DenmarkSøborg, Denmark; 7 Institute of Public Health, University of Southern DenmarkOdense, Denmark; 8 MRC Epidemiology UnitCambridge, United Kingdom

## Abstract

**Background:**

Although habitual physical activity energy expenditure (PAEE) and cardio-respiratory fitness (CRF) are now well-established determinants of metabolic disease, there is scarcity of such data from Africa. The aim of this study was to describe objectively measured PAEE and CRF in different ethnic populations of rural Kenya.

**Methods:**

A cross-sectional study was done among 1,099 rural Luo, Kamba, and Maasai of Kenya. Participants were 17–68 years old and 60.9% were women. Individual heart rate (HR) response to a submaximal steptest was used to assess CRF (estimated VO_2_max). Habitual PAEE was measured with combined accelerometry and HR monitoring, with individual calibration of HR using information from the step test.

**Results:**

Men had higher PAEE than women (∼78 vs. ∼67 kJ day^−1^ kg^−1^, respectively). CRF was similar in all three populations (∼38 and ∼43 mlO_2_·kg^−1^ min^−1^ in women and men, respectively), while habitual PAEE measures were generally highest in the Maasai and Kamba. About 59% of time was spent sedentary (<1.5 METs), with Maasai women spending significantly less (55%). Both CRF and PAEE were lower in older compared to younger rural Kenyans, a difference which was most pronounced for PAEE in Maasai (−6.0 and −11.9 kJ day^−1^ kg^−1^ per 10-year age difference in women and men, respectively) and for CRF in Maasai men (−4.4 mlO_2_·min^−1^ kg^−1^ per 10 years). Adjustment for hemoglobin did not materially change these associations.

**Conclusion:**

Physical activity levels among rural Kenyan adults are high, with highest levels observed in the Maasai and Kamba. The Kamba may be most resilient to age-related declines in physical activity. Am. J. Hum. Biol. 2012. © 2012 Wiley Periodicals, Inc.

Habitual physical activity and cardio-respiratory fitness (CRF) are important regulators of metabolism and major determinants of modern lifestyle diseases such as Type 2 diabetes and cardiovascular disease (Gill et al.,^2006^; Helmrich et al.,^1991^). Several studies have reported a reduction in CRF among ethnic groups in different continents as a consequence of a shift from traditional to more modern lifestyle (Celis-Morales et al.,^2011^; Ekblom and Gjessing,^1968^; Rode and Shephard,^1984^).

In East Africa, a secular increase in metabolic disease burden has been observed over recent decades, with diabetes prevalence of rural dwellers in Tanzania estimated at 0.9% in 1989 (McLarty et al.,^1989^) and 2.2% in rural Kenyan dwellers in 2009 (Christensen et al.,^2009^). Similarly, cholesterol levels in the Maasai have increased by about 12% over the last four decades (Biss et al.,^1970^; Mbalilaki et al.,^2010^). However, there is a paucity of data on free-living physical activity and CRF. Further, most epidemiological studies in African populations in which the association between physical activity and chronic diseases have been examined have used questionnaire-based exposure measurement (Aspray et al.,^2000^; Ezenwaka et al.,^1997^; Forrest et al.,^2001^; Kruger et al.,^2002^; Mbalilaki et al.,^2007^; Sobngwi et al.,^2002^). However, the validity of questionnaires to assess quantitative measures of physical activity such as its associated energy expenditure (PAEE) has been questioned (Wareham,^2001^).

Population-based free-living PAEE has only been objectively assessed in one African study, which was conducted in Cameroon (West Africa) using combined accelerometry and heart rate (HR) monitoring (Assah et al.,^2011^), a method which demonstrated good agreement with the doubly labeled water method (Assah et al.,^2011^). Rural dwellers of Cameroon were found to be more active than urban residents in both men (66.5 vs. 53.4 kJ day^−1^ kg^−1^) and women (55.7 vs. 38.9 kJ day^−1^ kg^−1^), a difference which was also reflected in CRF levels.

In East Africa, rural Maasai men were reported to have high CRF (=55 mlO_2_·kg^−1^ min^−1^), and markedly lower CRF from age 44 years onward (Mann et al.,^1965^). However, a similar age-related difference in CRF could not be found in rural Kenyan men and women of different ethnic groups (di Prampero and Cerretelli,^1969^). Both of these studies from the 1960s used objective methods to estimate CRF but were relatively small and, apart from reports in specific occupational groupings as for example sugarcane cutters in Tanzania (Davies and Van Haaren,^1973^), more recent population data are missing.

Rural East Africa is dominated by ethnic clustering and economies based on traditional lifestyle. Agriculture is the most widespread economy but some ethnic groups depend also on fishing or pastoralism for their livelihood (Hansen et al.,^2011^). In addition, these populations live at different altitude, and taken together these differences may result in diverse levels of fitness and physical activity.

The purpose of this study was to compare objective measures of CRF and physical activity, including PAEE, in three population-based samples of rural Luo, Kamba and Maasai in Kenya, representing predominantly agro-fishing, agricultural, and agro-pastoralist lifestyles, respectively.

## STUDY POPULATION AND METHODS

### Study area and population

Inclusion criteria for the study were age ≥17 years and Luo, Kamba, or Maasai ethnicity and living largely traditional lifestyles in rural districts of Kenya. Exclusion criteria were pregnancy, serious illnesses such as malaria, inability to walk unassisted and severe mental disease (Christensen et al.,^2008^; Hansen et al.,^2011^).

The Luo participants were examined at thirteen different primary schools in Bondo district around Lake Victoria ∼1,200 m above sea level (ASL). The Kamba participants were examined at Mutomo Hospital in Kitui District in eastern Kenya ∼1,700 m ASL. The Maasai participants were examined at Lolgorian Health Center in Transmara district ∼1,800 m ASL. All participants were volunteers who chose to participate following information given at local community meetings.

If the participant did not know his/her age, the local social mobilizer estimated his/her age according to personal events such as circumcision and age-set membership.

All participants gave written or oral informed consent. Ethical permission was obtained from the National Ethical Review Committee in Kenya and study approval was given by the Danish National Committee on Biomedical Research Ethics in Denmark.

Field work was completed between August and November 2005. A total of 1,178 individuals of mean (range) age 39.0 (17–68) years were included in the study, of whom 1,048 (89.0%) had complete information on CRF and 1,099 (93.3%) on physical activity. The ethnic distribution was 381 Luo (209 women, 172 men), 377 Kamba (283 women, 94 men), and 341 Maasai (178 women, 163 men).

### Assessment of physical activity and cardio-respiratory fitness

The physical activity and fitness measurements were carried out using a combined uniaxial accelerometer and HR sensor (Actiheart, Cambridge Neurotechnology, Cambridge, UK). Following an overnight fast, the monitor was applied to the chest on two ECG electrodes (Unomedical A/S, Birkerød, Denmark); a medial electrode placed at the lower part of the sternum, and a lateral electrode placed on the same horizontal level and as laterally as possible without stretching the wire below the major pectoral muscle (Brage et al.,^2006^).

Each participant was asked to perform an 8-min step test administered in the form of a drum beat included in the Actiheart software to facilitate time synchronization, stepping up and down a 21.5-cm high step (Reebok, Lancaster, UK). The stepping frequency was 15 step cycles (body lifts) per minute in the first minute, after which it increased linearly up to 33 steps per minute at the end. The test was followed by two minutes of sitting recovery. HR recovery was measured for at least 90 s after the step test, regressed against recovery time, and expressed as the HR above sleep at 1-min poststepping (Brage et al.,^2007^). The individual HR response to the step test was used to calibrate HR to protocol-estimated physiological intensity, and also to obtain an estimate of the CRF level (expressed as ml O_2_·min^−1^ kg^−1^) by extrapolation of the submaximal relationship to age-predicted maximal HR (Tanaka et al.,^2001^) and adding an estimate of resting metabolic rate (Henry,^2005^). For these analyses, only participants performing at least 4 min of stepping were included.

Following the step test procedure, the Actiheart was downloaded and reinitialized for habitual monitoring using 30-s epochs. The study participants were asked to wear the monitor continuously over the following 5 days, whilst continuing their normal daily activities. HR data were preprocessed using a robust Gaussian Process Regression method (Stegle et al.,^2008^). From these data, sleeping HR, average HR above sleep and body movement (accelerometry) during free-living, as well as physical activity phenotypes were derived. In particular, physical activity intensity was estimated by branched equation modeling (Brage et al.,^2004^) to combine step-test calibrated HR information with the accelerometry-based estimate of intensity (Brage et al.,^2007^). Average daily PAEE was derived by integration with respect to time, whilst minimizing potential diurnal information imbalance bias. Individuals were included in the present analyses if they contributed >24 h of valid (monitor worn) activity data.

Total energy expenditure (TEE) was derived by adding a component for resting metabolic rate (RMR) and a component for diet-induced thermogenesis (10% of TEE) to the average daily PAEE. RMR was estimated using age, sex, weight, and height (Henry,^2005^). Physical activity level (PAL) was calculated as the ratio of daily TEE/RMR, from which prevalence of physical inactivity was defined as proportion of individuals with a PAL < 1.6 (Shetty et al.,^1996^). The time distribution of activity intensity was described by first converting the intensity time-series to units of MET using the estimate of RMR. Sedentary behavior was then defined as intensity <1.5 MET, while moderate and vigorous physical activity (MVPA) was defined as intensity >3 MET. Time spent in these categories was then summarized across the time-series data for each individual.

Participants who completed <4 min of the step test were asked to do another step test after the monitoring period of habitual activity. For the present analysis, however, completion of >2 min of a step test was regarded as sufficient for inclusion, using a combination of slope-and-intercept and 1-point calibration methods (Brage et al.,^2007^). Specifically, we used the HR at the 2-min point during stepping to derive a 1-point model for fitness in the individuals providing >4 min of stepping; this was then applied in the individuals stepping between 2 and 4 min. Activity data from individuals who did not have a valid step test calibration were processed with a group-based equation which only takes gender, age, and sleeping HR of the individual into account, derived using all available valid step test data.

### Blood test

Blood hemoglobin (Hb, in g dl^−1^) was determined using a standard Coulter counter technique (model KX-21N, Sysmex Corporation, Kobe, Japan).

### Statistics

We used sleeping HR, step recovery HR, and estimated VO_2max_ as dependent CRF variables. Hb was used as dependent oxygen-binding capacity variable. Average habitual HR above sleep, accelerometric movement, time spent (%) <1.5 MET, time spent (%) >3 MET, PAEE and PAL were used as dependent physical activity variables. Ethnic grouping was treated as a categorical variable. Analysis of variance was used to test for differences in means and overall group effect, followed by Tukey post-hoc test for between-group differences. Means for dependent variables were standardized to 40 years of age by use of linear regression. Data for females and males were analyzed separately. Sensitivity analyses were conducted using stricter criteria for inclusion of fitness estimates (step test duration >4 min) and physical activity data (>72 h of valid data). *P*-values <0.05 were considered statistically significant. All analyses were done with the Stata 11.2 IC version (Stata, College Station, USA).

## RESULTS

Participant characteristics are shown in Table 1. Maasai participants were slightly younger, and the Kamba had the lowest weight and height. Hb level was lowest in the Luo and highest in the Maasai.

**TABLE 1:** Biological background characteristics stratified by ethnicity and gender

	Luo (*n* = 381)	Kamba (*n* = 377)	Maasai (*n* = 341)	*P* value^a^
Age (years)				
Women	39.0 (9.7)	40.3 (9.5)	34.6 (10.4)^b^,^c^	<0.001
Men	39.9 (10.5)	42.2 (10.5)	38.5 (11.6)^c^	0.029
Weight (kg)				
Women	58.1 (56.5; 59.6)	55.6 (54.2; 56.9)	59.2 (57.4; 60.9)^c^	0.004
Men	64.2 (62.5; 65.9)	56.0 (53.7; 58.4)^b^	64.0 (62.2; 65.8)^c^	<0.001
Height (cm)				
Women	163.2 (162.4; 164.0)	156.7 (156.0; 157.4)^b^	161.4 (160.5; 162.3)^b^,^c^	<0.001
Men	174.2 (173.2; 175.2)	167.9 (166.6; 169.3)^b^	174.2 (173.2; 175.2)^c^	<0.001
BMI (kg m^−2^)				
Women	21.8 (21.2; 22.4)	22.6 (22.1; 23.1)	22.7 (22.1; 23.4)	0.063
Men	21.1 (20.6; 21.6)	19.8 (19.1; 20.5)^b^	21.1 (20.5; 21.6)^c^	0.009
Hemoglobin (g dl^−1^)				
Women	12.1 (11.8; 12.3)	12.6 (12.4; 12.8)^b^	12.7 (12.4; 13.0)^b^	0.001
Men	14.1 (13.8; 14.3)	14.5 (14.2; 14.9)	15.7 (15.4; 15.9)^b^,^c^	<0.001

Values for age are mean (SD). Other values are mean (95% CI), standardized to 40 years of age.

a P value denotes overall difference between groups.

b Significantly different from Luo (*P* < 0.05) in Tukey post-hoc test (adjusted for age).

c Significantly different from Kamba (*P* < 0.05) in Tukey post-hoc test (adjusted for age).

The median (interquartile range) step test duration was 7.0 (5.0–8.0) min, with 1049 participants stepping >2 min and 904 stepping ≥4 min. The Actiheart was worn over an average (range) of 3.9 (1.0–8.1) days, slightly shorter among the Maasai (3.8 days) compared to the Luo (4.0 days, *P* = 0.050), and the Kamba (4.0 days, *P* = 0.032), with 1099 participants accumulating >24 h and 1,039 accumulating >72 h of valid activity data.

CRF and PAEE in the three ethnic groups are presented in Table 2. Kamba women had the highest sleeping HR. Maasai had the lowest and Luo the highest recovery HR (above sleep) in women and men, respectively but no differences were found in CRF between the three ethnic groups in neither gender. Similar patterns were observed when stricter criteria for inclusion (>4-min step test duration) were applied.

**TABLE 2:** Estimations of cardio-respiratory fitness and physical activity for ethnic groups by sex

	Luo (*n* = 381)	Kamba (*n* = 377)	Maasai (*n* = 341)	*P* value^a^
Sleeping HR (beats min^−1^)				
Women	59.1 (58.1; 60.1)	61.0 (60.1; 61.8)^b^	59.2 (58.1; 60.3)^c^	0.007
Men	54.4 (53.3; 55.5)	55.2 (53.7; 56.8)	55.6 (54.4; 56.7)	0.342
Recovery HR above sleep^d^ (beats min^−1^)				
Women	30.1 (28.0; 32.2)	27.7 (25.9; 29.5)	23.4 (21.1; 25.7)^b^,^c^	<0.001
Men	26.3 (24.3; 28.4)	20.9 (18.2; 23.6)^b^	21.4 (19.4; 23.4)^b^	<0.001
Cardio-respiratory fitness^e^ (ml O2 min^−1^ kg^−1^)				
Women	37.8 (36.8; 38.7)	37.7 (36.9; 38.5)	38.9 (37.8; 39.9)	0.212
Men	42.9 (42.0; 43.9)	43.1 (41.7; 44.4)	43.1 (42.1; 44.1)	0.971
Habitual HR above sleep (beats mini^−1^)				
Women	19.4 (18.9; 19.9)	20.4 (20.0; 20.9)^b^	21.2 (20.6; 21.8)^b^	<0.001
Men	20.1 (19.4; 20.7)	20.1 (19.3; 21.0)	19.1 (18.5; 19.8)	0.087
Accelerometric movement (m s^−2^)				
Women	0.13 (0.09; 0.17)	0.18 (0.15; 0.22)	0.15 (0.10; 0.19)	0.134
Men	0.17 (0.15; 0.18)	0.20 (0.19; 0.22)^b^	0.21 (0.20; 0.23)^b^	<0.001
PAEE^f^ (kJ day^−1^ kg^−1^)				
Women	58.9 (55.0; 62.9)	67.4 (64.0; 70.8)^b^	74.5 (70.1; 78.9)^b^,^c^	<0.001
Men	74.4 (70.8; 78.1)	80.9 (75.9; 85.9)	78.0 (74.2; 81.7)	0.110
PAL^f^ (TEE/RMR)				
Women	1.81 (1.76; 1.86)	1.90 (1.86; 1.94)^b^	1.99 (1.94; 2.05)^b^,^c^	<0.001
Men	1.93 (1.89; 1.96)	1.95 (1.90; 2.00)	1.95 (1.91; 1.99)	0.592
% Time spent <1.5 MET^f^				
Women	60.2 (58.8; 61.6)	59.2 (58.0; 60.5)	55.2 (53.6; 56.8)^b^,^c^	<0.001
Men	59.4 (57.9; 60.8)	57.7 (55.7; 59.6)	58.9 (57.5; 60.4)	0.381
% Time spent >3 MET^f^				
Women	9.0 (8.2; 9.9)	11.4 (10.7; 12.2)^b^	13.2 (12.3; 14.2)^b^,^c^	<0.001
Men	12.8 (11.9; 13.6)	13.3 (12.1; 14.5)	13.5 (12.6; 14.4)	0.501

Values are mean (95% CI), standardized to 40 years of age.

HR: heart rate; MET: metabolic equivalent; PAEE: physical activity expenditure; PAL: physical activity level; TEE: total energy expenditure; RMR: resting metabolic rate.

a *P*value denotes overall difference between groups

b Significantly different from Luo (*P* < 0.05) in post-hoc test (adjusted for age).

c Significantly different from Kamba (*P* < 0.05) in post-hoc test (adjusted for age).

d Recovery heart rate at 1-min poststep test (based on 90-s regression), expressed above sleep HR.

e Sample sizes are *n* = 367 (Luo), *n* = 358 (Kamba), and *n* = 324 (Maasai).

f Individually calibrated, except in those without valid step test where the following group calibration equation (based on all valid step tests) was used: Activity intensity [J min^−1^ kg^−1^] = (6.80 − 0.005 age + 0.30 gender − 0.0009 SHR) × HRaS − 0.02 age + 16.1 gender − 1.07 SHR − 11.

Accelerometric movement was lowest in Luo men and women but only in the women was this significantly reflected in PAEE, PAL, and proportion of time spent in MVPA. These measures also suggested that Maasai women were most physically active, and spent less time sedentary. Adjusting for Hb as a proxy for oxygen-binding capacity did not change the group mean estimates (data not shown); however, Hb was positively associated with CRF in women (*P* = 0.001) but not in men (*P* = 0.574), whereas men with higher Hb had higher PAEE (*P* = 0.038) but this association was not significant in women (*P* = 0.620).

CRF was positively correlated with PAEE (*r* = 0.59, *P* < 0.001); the relationship being a 1.9 kJ day^−1^ difference in PAEE for every 1 ml O_2_ min^−1^ difference in CRF (similar in men and women and across ethnic groups).

Using PAL <1.6 to denote prevalence of physical inactivity and adjusted to 40 years of age using logistic regression, mean (95% CI) prevalence was highest in Luo women (23.1%, 16.3; 32.7%), followed by the Kamba (13.2%, 9.1; 18.9%) and the Maasai (6.3%, 3.3; 12.0%). In men, corresponding estimates were 12.8% (7.9; 20.7%) in Luo, 8.6% (4.2; 17.4%) in Kamba, and 6.9% (3.6; 12.9%) in Maasai. Using stricter criteria for inclusion (>72-h activity data) did not materially change observed differences between ethnic groups.

**Fig. 1:**
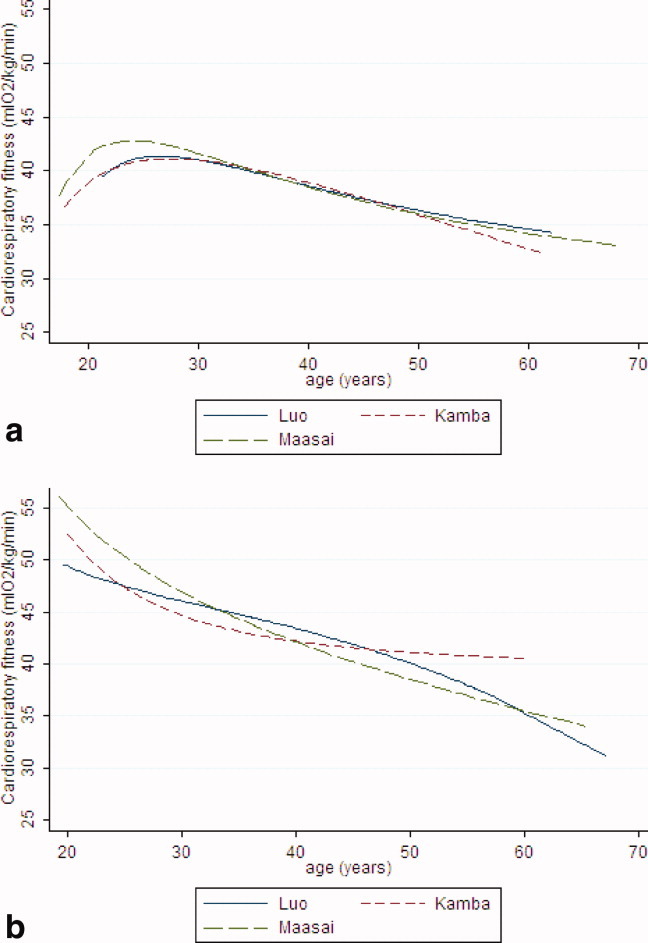
(a) The relationship between cardio-respiratory fitness (ml min^−1^ kg^−1^) and age among women, stratified by ethnic group (polynomial regression lines). For reference, a decline of ∼2 ml O2/10 years denote the expected difference in CRF induced by using age-estimated maximal HR. (b) The relationship between cardio-respiratory fitness (ml min^−1^ kg^−1^) and age among men, stratified by ethnic group (polynomial regression lines). For reference, a decline of ∼2 ml O2/10 years denote the expected difference in CRF induced by using age estimated maximal HR.

**Fig. 2:**
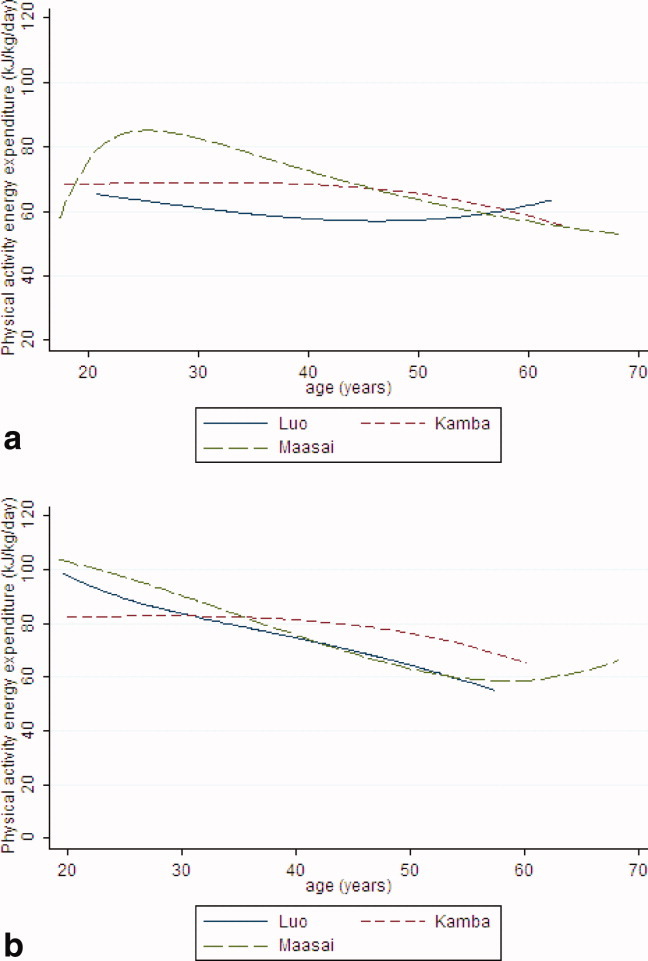
(a) The relationship between activity energy expenditure (kJ day^−1^ kg^−1^) and age among women, stratified by ethnic group (polynomial regression lines). (b) The relationship between activity energy expenditure (kJ day^−1^ kg^−1^) and age among men, stratified by ethnic group (polynomial regression lines). [Color figure can be viewed in the online issue, which is available at wileyonlinelibrary.com.]

CRF as well as PAEE were inversely associated with age in all three ethnic groups (Figs. 1 and 2). For each 10-year difference in age, CRF was about 2 mlO_2_·min^−1^ kg^−1^ lower in women, while in the men (with over 10% higher mean levels), the age-related differences were of similar magnitude in Kamba but more pronounced in Luo and Maasai (−3.0 and −4.6 mlO_2_·min^−1^ kg^−1^ per 10 years, respectively) men. For PAEE, age-related differences were highest in the Maasai at −6.0 and −11.9 kJ day^−1^ kg^−1^ per 10 years in women and men, respectively (Table 3).

**TABLE 3:** Age-related differences in cardio-respiratory fitness and physical activity energy expenditure for ethnic groups by sex

	Women	*P* value	Men	*P* value
CRF^a^ (ml O2 min^−1^ kg^−1^)				
Luo	−1.6 (−2.6; −0.7)	0.001	−3.0 (−4.0; −2.1)	<0.001
Kamba	−1.9 (−2.9; −1.0)	<0.001	−1.9 (−3.2; −0.4)	0.001
Maasai	−2.1 (−3.0; −1.2)	<0.001	−4.4 (−5.2; −3.6)	<0.001
PAEE^b^ (kJ day^−1^ kg^−1^)				
Luo	−1.9 (−4.7; 1.0)	0.204	−10.1 (−13.7; −6.5)	<0.001
Kamba	−2.0 (−6.5; 2.6)	0.391	−3.9 (−9.5; 1.7)	0.169
Maasai	−6.0 (−9.4; -2.7)	<0.001	−11.9 (−14.8; −9.0)	<0.001

All data are mean differences (95% CI) per 10-year difference in age. For reference, a decline of ∼ 2 ml O_2_/10 year (∼ 0.29 mlO_2_·kg^−1^ beat^−1^ × 7 beat/10 year) denotes the expected difference in CRF induced by using age-estimated maximal HR.

a CRF: cardio-respiratory fitness (*N* = 629 women, *N* = 420).

b PAEE: physical activity energy expenditure (*N* = 670 women, *N* = 429 men).

## DISCUSSION

We observed relatively low levels of CRF in men (∼43 mlO_2_·kg^−1^ min^−1^) and women (∼38 ml O_2_·kg^−1^ min^−1^), respectively, among all three ethnic groups (adjusted to 40 years of age). In comparison with earlier studies of rural populations, and keeping method differences in mind, the Maasai had 10–25% higher CRF levels four decades earlier for the same age (di Prampero and Cerretelli,^1969^; Mann et al.,^1965^). However, the average CRF level of the Kamba in our study was only 5% lower, compared to the biologically and culturally related Kikuyu and Meru measured in the 1960s (di Prampero and Cerretelli,^1969^). Current fitness levels are also lower than those measured in Tanzanian sugarcane cutters in the 1970s (Davies,^1973^; Davies and Van Haaren,^1973^). Thus, the overall trend suggests a decline in fitness of rural East Africans over time and for the Maasai population in particular.

Bearing in mind that age differences in CRF estimated from submaximal tests should be interpreted with caution, the age-related difference was evident in all three ethnic groups but more pronounced in men compared to women, and especially in the Maasai. A similar inverse relationship between CRF and age was observed in rural Maasai from Tanzania four decades earlier (Mann et al.,^1965^). Culturally determined changes in physical activity with increasing age as well as age-related sarcopenia are possible explanations for what seems a fairly large difference in CRF between young and old adults.

Age-related differences in prevalence of overweight and obesity in the same study population (Christensen et al.,^2008^) may at least partly explain the parallel differences in CRF levels. These findings are also in line with our previous observations on lower glucose tolerance in older adults, although the rural population as a whole has a relatively low diabetes prevalence of 2.2% (Christensen et al., 2009). This is, however, still higher than earlier estimates of 0.9% (McLarty et al.,^1989^), which is paralleled by a secular increase in population cholesterol levels (Biss et al.,^1970^; Mbalilaki et al.,^2010^). This places the suggested decline in physical activity and fitness over time in a noncommunicable disease context.

Sleeping HR and recovery HR also reflect CRF in an inverse manner (Brage et al.,^2007^; Darr et al.,^1988^). In the present study, recovery HR was highest in the Luo among both women and men, while sleeping HR was similar among the three ethnic groups. CRF is to some extent dependent on the Hb level, which in turn depends on iron status and intake of other micronutrients, history of inflammation, as well as exposure to altitude. These environmental influences were reflected in the Hb values in men and women with a tendency among the Maasai to have the highest and the Luo to have the lowest values. Given that CRF levels were similar within each gender in the three ethnic groups, this may reflect that fitness-enhancing activity is lower in the Maasai population and higher in the Luo.

A recent study found a strong dose-response relationship between volume of exercise and change in CRF (Church et al.,^2007^), and in the current study a strong cross-sectional relationship was also observed between PAEE and CRF. However, increasing physical activity levels will not necessarily increase CRF if the activity is predominantly of low intensity (Gill,^2007^). We measured PAEE over ∼4 days and expressed it relative to RMR to get an impression of the relative energy cost of daily activities. These results showed that the men spent roughly 13% of their time, or about 3 h day^−1^, in MVPA (>3 METs). The same was true for the Maasai women and MVPA was still over 2 h day^−1^ in the Luo women, who were the least active. This suggests that the intensity distribution of habitual physical activity in rural Kenyans of today may be different than it was decades ago.

We also observed lower PAEE with in older compared to younger in both genders. As for CRF, the difference was most pronounced in the Maasai, and more so in men compared to women. These differences point towards more pronounced age-related and gender-specific cultural changes in the Maasai, compared to the Luo and Kamba people.

An accelerometer on the trunk as was used in the present study cannot meaningfully measure physical activity while an individual is carrying out work with his or her arms (Hendelman et al.,^2000^) but picks up walking very well (Brage et al.,^2007^). The lower values obtained in accelerometric movement in the Luo compared to the Kamba and Maasai participants may therefore be a result of differences in mode of physical activity with more walking taking place among the Kamba and Maasai, as PAEE was higher in these populations.

In general, the PAL values have to be interpreted with caution in this study, as RMR and dietary-induced thermogenesis are both estimated. Of particular note is RMR, which has been estimated using only participants' age, sex, height, and weight (Henry,^2005^). According to Henry and Rees (Henry and Rees,^1991^), there is some evidence of an overestimation of basal metabolic rate in people from tropical countries in general by using estimated values obtained from mainly other population groups. Basal metabolic rate was overestimated by 6.5% in African males—no data exist on African females—when compared to the Schofield equations (Schofield,^1985^), and the newer equations only make up for part of this discrepancy (Henry,^2005^). A similar bias would impact on amount of time spent between <1.5 METs and >3 METs.

The present study showed no difference in the prevalence of physical inactivity (PAL < 1.6) between the ethnic groups among the men. However, Maasai women had the lowest and the Luo women the highest prevalence of physical inactivity. These dichotomized data correspond well with the mean values for PAEE, especially in the women.

The average PAL values in the present study populations ranged from 1.81 to 1.99. In contrast, the energy expenditure of Gambian women and men measured during peak agricultural activity using the doubly labeled water method and HR measurement showed a mean PAL value >2.3 in nonpregnant women (Singh et al.,^1989^) and 2.4 in men (Heini et al.,^1996^). Compared with our results this dramatic difference may indicate a difference in activity levels as a result of seasonal variation as we collected data following but not during the harvest season. Nonetheless, these activity levels from rural East African populations are 15–20% higher than recent estimates from rural West Africans (Assah et al.,^2011^) and >50% higher than levels measured in 55-year-old Europeans (Eur J Epidemiol,^2012^), both studies using the same assessment method for physical activity. To obtain a more accurate picture of habitual PAEE, future work should adopt a repeated measurement design to reflect possible seasonal variation.

The lower average PAL among the women compared to the men (except in the Maasai) may be a result of an energy-saving mechanism, and thus not necessarily less physical activity *per se* as Maloiy et al. (1986) found low energy costs of carrying heavy loads on the heads among Luo and Kikuyu women. These findings have been confirmed later by others (Cavagna et al.,^2002^; Heglund et al.,^1995^). To some extent, similar results have been found in Gambian men, who had a 3% greater net efficiency of walking at level and 10% elevation on a treadmill compared to European men (Minghelli et al.,^1990^). Nevertheless, the combined overall lower accelerometric movement, lower PAEE and PAL measurements in women provide some evidence that the females in our study were less physically active than the males.

No questionnaire-based studies on physical activity in African populations have investigated ethnic differences (Aspray et al.,^2000^; Ezenwaka et al.,^1997^; Mbalilaki et al.,^2007^; Sobngwi et al.,^2002^) but confirm our findings that men are more physically active than women (Ezenwaka et al.,^1997^; Kruger et al.,^2002^).

Certain limitations of this study should be acknowledged. First of all, potential selection bias cannot be ruled out due to not being able to use a random sampling frame. Second, the key variables, CRF and PAEE, although based on objective measurements, are still the results of estimations by modeling; they are not measured directly.

In conclusion, this is the first epidemiological study to objectively measure habitual physical activity in East African populations. In combination with other changes in lifestyle, age-related declines in physical activity and CRF, especially in the Maasai and in the men, this may increase the risk of noncommunicable diseases in rural populations of low income.
